# Rheology of Poly(glycidyl methacrylate) Macromolecular Nano Assemblies

**DOI:** 10.3390/polym14030455

**Published:** 2022-01-23

**Authors:** Andrés Cardil, Miguel Palenzuela, Juan F. Vega, Marta E. G. Mosquera

**Affiliations:** 1 Departamento de Química Orgánica y Química Inorgánica, Instituto de Investigación en Química “Andrés M. del Río” (IQAR), Campus Universitario, Universidad de Alcalá, Alcalá de Henares, 28871 Madrid, Spain; andres.cardil@iem.cfmac.csic.es (A.C.); miguel.palenzuela@uah.es (M.P.); martaeg.mosquera@uah.es (M.E.G.M.); 2 BIOPHYM, Departamento de Física Macromolecular, Instituto de Estructura de la Materia, IEM-CSIC, c/Serrano 113 bis, 28006 Madrid, Spain

**Keywords:** poly(glycidyl methacrylate), macromolecular nanoassembly, fragility, linear viscoelasticity

## Abstract

A recently reported combined polymerization process of glycidyl methacrylate, mediated by homometallic and heterobimetallic aluminium complexes, naturally produces nano-sized macromolecular assemblies. In this work, the morphological features and the rheological properties of these novel nanoassemblies are studied. The hydrodynamic sizes of the nanoparticles in the solution range from 10 to 40 nm (in numbers), but on a flat surface they adopt a characteristic thin disk shape. The dynamic moduli have been determined in a broad range of temperatures, and the time—temperature superposition applied to obtain master curves of the whole viscoelastic response from the glassy to the terminal regions. The fragility values obtained from the temperature dependence are of m ~40, typical of van de Waals liquids, suggesting a very effective packing of the macromolecular assemblies. The rheological master curves feature a characteristic viscoelastic relaxation with the absence of elastic intermediate plateau, indicating that the systems behaved as un-entangled polymers. The analysis of the linear viscoelastic fingerprint reveals a Zimm-like dynamics at intermediate frequencies typical of unentangled systems. This behaviour resembles that observed in highly functionalized stars, dendrimers, soft colloids and microgels.

## 1. Introduction

Glycidyl methacrylate (GMA), is a monomer that deserves a great deal of industrial interest as it presents two functional groups. Monomers such as GMA, with two reactive groups, are very valuable as precursors of functionalized polymers when only one group is polymerized. Functional polymers are now a focus of research interest mainly due to their versatility. They can be used, for example, for the construction of blocks and new architectures and molecular topologies, including stars, dendrimers and networks or lattices. This versatility is a great advantage in the search for uses related to added value applications in recyclability of plastics (compatibilizers and tie layers), drug release systems, materials with shape memory effects, biocompatible agents, biomedicine, etc. [1,2]. We have recently developed a novel method to obtain new polymeric macromolecular assemblies by means of covalent crosslinking of a network of combs obtained from the ring opening polymerization of GMA [3,4]. The obtained nanoassemblies are soft nanoparticles of crosslinked poly(glycidyl methacrylate), cPGMA. These macromolecular structures provide a rechargeable substrate for an antimicrobial coating that shows resistance to bacterial colonization [5], and it can be used in an extensive range of biomedical applications, similarly to other acrylate based thermoresponsive polymers [6]. The approach is similar to that reported previously for stimuli sensitive polyglycidol, poly(*N*-isopropylacrylamide), poly(*N*-vinylcaprolactam), and poly (vinyl alcohol) microgels [7,8,9,10].

The rheological properties of soft nanoparticles were studied in the past in the pioneering works of Antonietti et al. [11,12]. They observe viscous flow for such microgels with diameters of up to 20 nm. The typical Zimm scaling of hydrodynamically coupled systems, G* ~ ω^2/3^, is also observed, but as these spherical particles can neither reptate nor perform Zimm movements, the mobility was explained as due to cooperative movements of coupled chains. Moreover, it was also envisaged that the mobility was effectively modulated not only by the size but also by the cross-linking density of the nanoparticles [13]. Besides the soft nanoparticles and microgels, a variety of macromolecular architectures, characterized by the lack of entanglements in the melt, has shown a similar rheological behaviour in the linear region. Examples are dendrimers [14,15,16,17], bottlebrushes [18,19,20,21], high functionalized stars [12,13,14,15,16,17,18,19,20,21,22,23,24,25] and dendronized polymers [26,27,28]. All these materials share certain rheological features as Rouse or Zimm-like dynamics, low values of the viscosity and high elastic character. Interestingly, the main feature shared in all the cases is the absence of entanglements. In all these cases, the construction of the macromolecular ensembles gives rise to certain topological and structural characteristics that facilitate the macromolecular crowding at the periphery and prevents the formation of entanglements. This leads to the absence of reptation mechanisms for relaxation and gives rise to the appearance of Rouse or Zimm-like dynamics, even in samples with very high molecular weights. Of course, the topological aspects of the specific macromolecular assembly modulate the physical properties and final applications as photonic crystals, drug delivery, supersoft elastomers, surfactants and rheological modifiers or lubricants.

In this work, we studied novel polymeric macromolecular assemblies of soft nanoparticles prepared by a new approach for covalent crosslinking of the network of combs obtained from the ring opening polymerization of GMA. The assemblies were characterized by dynamic light scattering, transmission electron and atomic force microscopies and rheology. The soft nanoassemblies have shown an intermediate fragility, far from that observed for amorphous polymers but close to that typical of strong liquids. The analysis of the linear viscoelastic fingerprint reveals specific Zimm-like dynamics at intermediate frequencies typical of unentangled systems. This behaviour resembles that observed in dendrimers, highly functionalized stars, soft colloids and microgels.

## 2. Materials and Methods

### 2.1. Materials

GMA, toluene, methanol and chloroform were obtained from Sigma-Aldrich (St. Louis, MO, USA). All manipulations were carried out under an inert atmosphere of Argon using standard Schlenk and glovebox techniques. All solvents were dried prior to use following standard methods. GMA was purified by vacuum distillation with CaH_2_ and stored under Argon. All reagents were commercially obtained and used without further purification. [AlXMe{2,6-(CHPh_2_)_2_-4-^t^Bu-C_6_H_2_O}]_n_ (X = Me, Cl), and [M{2,6-(CHPh_2_)_2_-4-^t^Bu-C_6_H_2_O}]_n_ (M = Li, Na, K) were prepared according to reported methods [3,4].

### 2.2. Synthesis

**Scheme 1:**
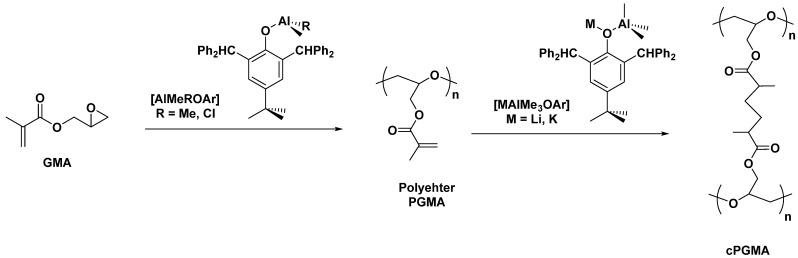
Combined polymerization of GMA with homometallic and heterometallic aluminium catalysts.

The nanoassemblies cPGMA1-3 were prepared through the same strategy that consisted of the combined polymerization of GMA with two different aluminium complexes as catalyst. In the first step, GMA is reacted with a 1 mol% of an aluminium homometallic catalyst [AlMeXOAr] (X = Me, Cl) (OAr: {2,6-(CHPh_2_)2-4-^t^Bu-C_6_H_2_O}), followed by the addition of 1 mol% of an heterometallic aluminium and alkaline metal aluminate [MAlMe_3_OAr] (M = Li, K) (Scheme 1).

In a typical procedure, the GMA monomer was purified by vacuum distillation using CaH_2_. Once purified, it was stored at −20 °C in the absence of light under Argon. The polymerizations were performed inside the glovebox. In a 50 mL Teflon valvulated flask, the homometallic aluminium catalyst was placed (0.015 g—27.86 μmol—of [AlMe_2_OAr] for cPGMA1 and cPGMA3, 0.016 g—27.86 μmol—of [AlMeClOAr] for cPGMA2), and then 0.38 mL—2.786 mmol—of GMA added. After 30 min stirring at RT, the corresponding amount of the heterometallic complex was added to the flask (0.016 g—27.86 μmol—of [LiAlMe_3_OAr] for cPGMA1 and cPGMA2, 0.017 g— 27.86 μmol) of [KalMe_3_OAr] for cPGMA3) and then was heated at 100 °C for 7 h. The polymerization was quenched by the addition of 15 mL of methanol. After 2 h stirring, the polymer was filtered and then dried under vacuum at 70 °C overnight. NMR spectra were recorded at 400.13 (^1^H), 155.50 (^7^Li) and 100.62 (^13^C) MHz on a Bruker AV400. IR spectra have been obtained in a Perkin-Elmer FT-IR Spectrometer Frontier, recording the area between 4000 and 400 cm^−1^. The crosslinked poly(glycidyl methacrylate) obtained was confirmed by NMR and IR as described previously by us [4]. The difference in the synthesis for the studied cPGMA relies on the combination of the catalysts used as shown in Table 1.

### 2.3. Dynamic Light Scattering: Hydrodynamic Properties

Hydrodynamic size of the samples was obtained by means of Dynamic Light Scattering (DLS) using the Zetasizer Nano ZS (Malvern Instruments, Worcestershire, UK) at T = 293 K in a 12 μL quartz batch cuvette (ZEN2112). The results of the scattered intensity time correlation function, [*g*_2_(*t*)−1], were obtained for filtrated solutions of the cPGMA samples in chloroform at variable concentration (c < 10 mg·mL^−1^), every 10 s, with 15 acquisitions for each run. The sample solutions were illuminated by a λ_0_ = 633 nm laser, and the intensity of scattered light was measured by an avalanche photodiode at an angle of θ = 173°. The normalized time correlation function of the electric field *g*_1_(*τ*) can be obtained from the Siegert equation [29]:

(1) $$g_{2}\left(\tau \right)=B+\beta {\left[g_{1}\left(t\right)\right]}^{2}\;,$$

where *τ* is the lag time, *B* is the baseline and *β* (≤1) is a coherence factor that accounts for deviations from the ideal correlation and the experimental geometry. The cumulant analysis has been applied by a non-linear fitting procedure using the program SEDFIT (from https://spsrch.cit.nih.gov/software/ accessed on 19 January 2022) [30,31]. The corresponding cumulant for the field autocorrelation function is:

(2) $$g_{1}\left(\tau \right)=e^{-\Gamma t}\left[1+\frac{\mu_{2}\tau^{2}}{2}\right]\;,$$

where Γ = *D_z_*·q^2^ is the average decay rate with *D_z_* being the z-average diffusion coefficient and q = (4π*n*/λ_0_) sin(θ/2) corresponding to the magnitude of the scattering vector, *n* the refractive index of the solution, taken as that of the solvent (1.448 for chloroform), λ_0_ the wavelength of the laser, and θ the scattering angle. In Equation (2), *μ*_2_ is the variance of the distribution, and the polydispersity index is defined as Q = *μ_2_*Γ^−2^. Once the decay rate Γ is obtained, it is used to obtain *D_z_* and the size by taking advantage of the Stokes-Einstein relationship that relates Γ and, alternatively, the diffusion coefficient to the hydrodynamic radius, *r_h_*, as:

(3) $$r_{h}=\frac{k_{B}T}{6\pi \eta D_{z}}$$

In Equation (3), *k_B_* is the Boltzmann’s constant, *T* is the absolute temperature, and *η* is the solvent viscosity, *η* = 0.056 cP. The apparent molecular weight of the samples was obtained from their *r_h_* values relative to linear polystyrene standards.

### 2.4. Atomic Force and Transmission Electron Microscopies: Morphological Aspects

Atomic Force Microscopy (AFM) topography images were obtained in a μTA™ 2990 Micro-Thermal Analyser (TA Instruments, Inc., New Castle, DE, USA) in contact mode. The samples were prepared by deposition of a drop of the dilute CHCl_3_ solution onto glass wafers and the solvent was left to evaporate for 24 h. A V-shaped silicon nitride probe with a cantilever length of 200 μm and a spring constant of 0.032 N·m^−1^ was used. The size of the images was 5 μm^2^. A JEM-2100 (JEOL Ltd., Tokyo, Japan) transmission electron microscope (TEM) operated at 200 kV and a nominal magnification of 20,000 was used for bright field image studies. Digital images were recorded using an Orius Gatan CCD camera. The samples dispersed in chloroform solution were deposited onto 200 mesh carbon-coated copper grids and left in the air to dry for their examination.

### 2.5. Rheological Analysis: The Linear Viscoelastic Fingerprint

Small amplitude strain sweeps and frequency sweeps were carried out in order to locate the linear viscoelastic region and to determine the basic linear viscoelastic properties of the samples. The experiments were performed with a Bohlin CVO stress—controlled rheometer in a range of temperatures using a parallel plates geometry (15 mm diameter). The samples were allowed to equilibrate for at least 5 min at each temperature. First, the linear viscoelastic region has been located by measuring the dynamic moduli, G′ and G″, as a function of strain amplitude at a frequency of 1 Hz. Then, frequency sweeps in the strain—controlled mode at the strains selected (below 0.1), to ensure that the sample response remained in the linear region and then the structure was not destroyed during the experiment. The dynamic properties storage and loss moduli, G′ and G″, were determined by sweeping across the frequency range from 0.02 to 20 Hz. The measurements were performed within the temperature range 293 to 363 K, and finally the time-temperature superposition principle was applied.

## 3. Results and Discussion

### 3.1. Characterization of The Samples

#### 3.1.1. Hydrodynamic Size and Molecular Weight

**Figure 1:**
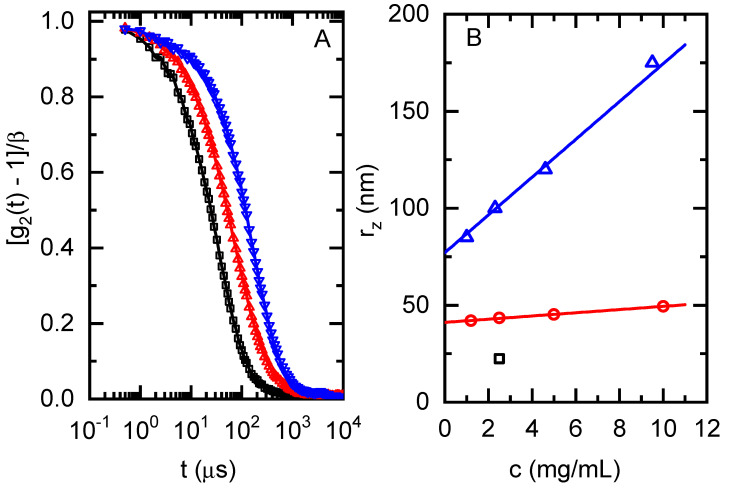
(**A**) Light intensity autocorrelation function, [*g*_2_(*t*)-1], of the samples under study at T = 293 K and c = 2.1 mg·mL^−1^versus time: (☐, black) cPGMA1, (Ο, red) cPGMA2, and (Δ, blue) cPGMA3. The lines represent the cumulant fits given by Equation (2) of the experimental results. (**B**) Intensity averaged radius of gyration, r_z_, as a function of concentration. Symbols as in Figure 1A.

DLS results are presented as the light intensity autocorrelation function, [*g*_2_(*t*)−1]. Figure 1A shows the values obtained for this function for the samples at T = 293 K and c = 2.1 mg·mL^−1^. A clear difference in [*g*_2_(*t*)−1] is observed in the samples under study. The results shown in this figure indicate a considerable difference in size among the samples studied, being cPGMA1 sample the smallest one and cPGMA3 the biggest. The cumulant analysis given by Equation (2) is suitable for obtaining the mean sizes. The analysis has been performed by serial dilution from 10 to 1 mg·mL^−1^, as it is observed in Figure 1B.

In Table 2, the extrapolated value to c = 0 mg·mL^−1^ is shown for each sample. The apparent molecular weight of the samples was determined by direct comparison of the experimental value of r_z_ with those obtained for PS standards. The results obtained are also listed in Table 2. The size distribution as numbers was also estimated in order to compare the results with those directly obtained by AFM and TEM analysis (see below). The values of the average values of r_n_ are also listed in Table 2. As usual in polydisperse systems, the number average hydrodynamic size is substantially lower than that obtained from the intensity average [32].

#### 3.1.2. Morphological Features of the Samples

**Figure 2:**
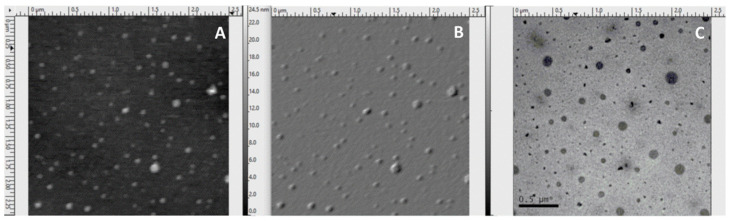
(**A**) Top-view and (**B**) Shaded-view of AFM image of cPGMA1 sample. (**C**) TEM image of cPGMA1 sample.

The results obtained by AFM and TEM for the cPEGMA1 sample are shown in Figure 2. The images reveal confident uniformity of the assemblies adsorbed onto the glass surfaces and the carbon coated grids. It is noteworthy that the relatively narrow height distribution obtained in AFM indicates that mostly individual cPGMA assemblies are present, and no agglomeration occurs in the conditions used for the preparation of the samples.

**Figure 3:**
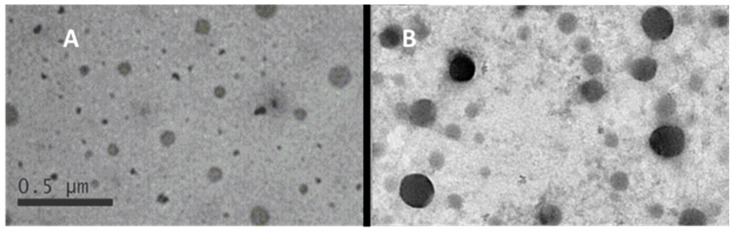
Comparison of the TEM images obtained for (**A**) cPGMA1 and (**B**) cPGMA3 samples. The bar corresponds to a size scale of 0.5 μm.

**Figure 4:**
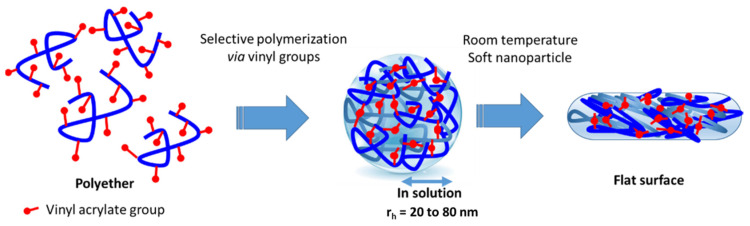
Schematic representation of the macromolecular assembly process, which is similar to those referred in [8,10].

Figure 3 compares the size of the particles obtained for cPGMA1 and cPGMA3 samples. The values of the size and molecular weight obtained for the systems under study indicate an intramolecular reaction that gives rise to relatively dense macromolecular assemblies. The scheme shown in Figure 4 illustrates the process of formation of the nano-sized particles from the linear precursors via covalent bonding [8,10]. The average radius (28.5 nm) obtained from the analysis of Figure 2A,B for cPGMA1 is much larger than the average height (6.5 nm) on the glass wafer surface [4]. These results point to the macromolecular assemblies spread during the adhesion process onto the glass wafer prior to the experiments, which is clearly indicative of their flexibility (see scheme in Figure 4).

The assemblies seem to adopt a pancake-like shape, a morphology already reported in dendrimers [33,34], flexible nanoparticles and nanogels [35,36,37,38]. Additional evaluation of the synthesized nanoassemblies by TEM in Figure 2C and Figure 3 confirms the morphology observed by AFM. The size observed is clearly higher than those obtained from the DLS experiments in numbers (r_n_). The result agrees with that recently reported by AFM in a condensed film for cPGMA3, with an average radius of around 80–100 nm and heights of 23 nm [5]. The experimental observations point to a controlled crosslinking process of GMA combs precursors into specific 3D assemblies. The range of sizes measured by DLS and TEM is relatively broad, and depends on the reaction conditions. This variability demonstrates the versatility of the approach to prepare systems of different sizes.

As described previously by us [3], in the ROP of GMA with homometallic derivatives [AlMeXOAr], different molecular weights were obtained depending on X. For X = Me, higher M_w_ (33.8 KDa) were obtained than for X = Cl (20 KDa). However, as shown in Table 3, the size of the final crosslinked polymer (cPGMA) is mostly affected by the alkaline metal present in the heterometallic catalyst employed for the crosslinking process. As such, when using the potassium aluminate [KAlMe_3_OAr] polymers with much higher size and molecular weight are formed than when using the lithium one. Interestingly, when using the same heterometallic aluminate, [LiAlMe_3_OAr], higher molecular weights are obtained for the shorter polymer precursor, which could be attributed to an easier access to the acrylate groups where the crosslinking reaction takes place.

### 3.2. Temperature Dependence of the Rheological Response: Fragility

**Figure 5:**
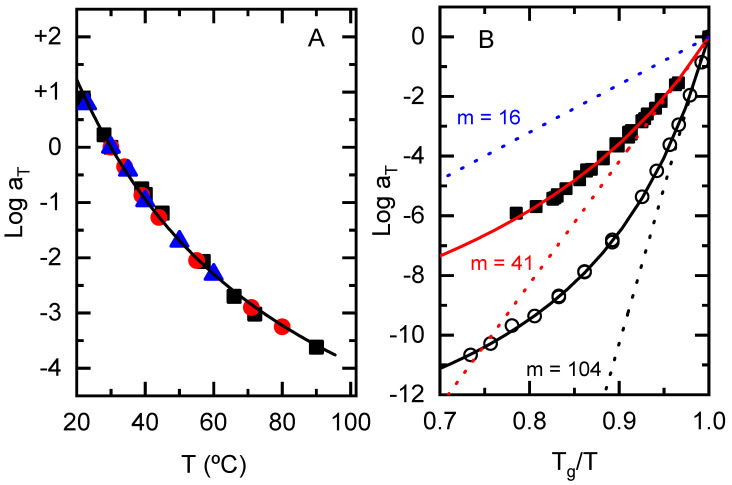
(**A**) Temperature dependence of horizontal shift factors, *a_T_*, for the samples studied. (■, black) cPGMA1, (●, red) cPGMA2, and (▲, blue) cPGMA3. The solid line is the WLF fit (Equation (4)) to experimental data. (**B**) Angell’s plot for the systems under study in comparison to the literature results: (■) cPGMAs, (Ο) amorphous PS.

Dynamic shear moduli, G ′(ω) and G ″(ω), master curves can be constructed by shifting the dynamic data obtained at each temperature to a reference, *T_R_*, based on the time−temperature superposition (TTS) principle. The results obtained for the cPGMA samples studied have been shifted to *T_R_* = 303 K. The horizontal shift factors, *a_T_*, have been plotted in Figure 5A, and the results can be fitted to the Williams−Landel−Ferry (WLF) equation:

(4) $$\log a_{T}=\frac{-C_{1}\left(T-T_{R}\right)}{C_{2}+\left(T-T_{R}\right)}$$

All the samples conform to the same dependence, with the values *c*_1_ = 9.7 C and *c*_2_ = 96.2, given by the solid line in Figure 5A. This behaviour implies similar values of *T_g_* for the samples (see Table 2). The measured values of *T_g_* were around 285 K for cPGMA1 and cPGMA3 samples. The reader is referred to our previous works in these samples for information about differential scanning calorimetry (DSC) characterization [4,5]. Glass-forming liquids, including polymers, exhibit strong non-Arrhenius temperature dependence of the temperature shift factors (relaxation time and viscosity) as observed in these materials. The variation with temperature of these properties can be quantified by the so-called fragility index, m [39,40,41], which can be defined at *T_g_* as:

(5) $$m={\frac{\partial loga_{T}}{\partial \left[\frac{T_{g}}{T}\right]}|}_{T=T_{g}}$$

The Angell’s plot in Figure 5B is very convenient to compare the results obtained in the cPGMA samples with those typical of amorphous polymers as polystyrene (PS), in terms of their fragility. The dynamic fragility for cPGMA samples was calculated from Equation (5). The results are clearly located in the intermediate region in between hard/fragile materials (characterized by high values of m ~ 150–200) and soft/strong materials with an Arrhenius behaviour (theoretical lower limit of m = 16) [42]. The value for the fragility obtained in cPGMA samples is m ~ 41, which is very close to that obtained in flexible polymers such as PIB, PE and PTHF [43,44]. Such low values of m, but still higher than those obtained here for cPGMA, have been reported in different types of dendronized polymers [26,27,28] and microgels [45], for which values of fragility between m = 53 and m = 75 have been measured. The Angell plot in Figure 5B clearly indicates that the cPGMA nano-sized objects present an intermediate behaviour between hard/fragile glasses and soft/strong colloids. What this means is that the cPGMA nanoparticles are able to accommodate their shape or conformation, reaching a more densely packed (stronger) than fragile polymers near *T_g_*.

Under the light of the recent model presented by van der Scheer et al. [45], the cPGMA nanoparticles, as soft compressible systems, might regulate their shape and volume in order to reach an osmotic equilibrium, with the elastic energy acting as effective fragility order parameter. On the contrary, high-fragility systems, as for example amorphous polystyrene (PS) have rigid polymeric chain and/or side groups. This rigidity makes efficient packing quite challenging, leading to a higher fragility. The softness of the cPGMA assemblies studied here anticipates characteristic rheological long-time dynamics. In the next section, we have explored the rheological response of the condensed films of the flexible nanoassemblies described in the preceding lines. The investigation of the impact of the hard/fragile character modulation on the structure and flow behaviour of condensed dispersions such as those studied here is of great interest. It is noteworthy that the structural features dictate basic physical properties, and determine their connections with applications, for example in pharmacy or biotechnology [45].

### 3.3. Linear Viscoelastic Response of the cPGMA Samples

**Figure 6:**
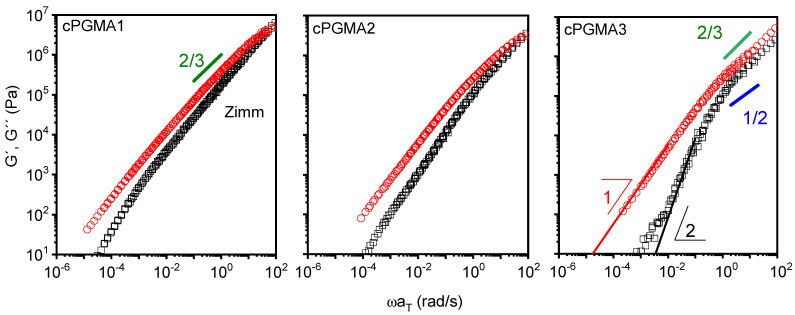
Dynamic master curves of G′ (squares) and G″ (circles) at a reference temperature *T_R_* = 303 K. Lines showing the slopes 1/2 (Rouse) and 2/3 (Zimm) are indicated.

Figure 6 shows the master curves of the storage, G′, the loss, G″, moduli of samples cPGMA 1, cPGMA2 and cPGMA3 at *T_R_* = 303 K. The superposition is excellent in all cases. The samples look very similar to the expected behaviour observed in unentangled polymers. In fact, the linear viscoelastic fingerprint resembles that predicted by the Rouse and Zimm models for unentangled systems [46,47], but being closer to the last one. Rouse dynamics predicts a power law of G* ~ ω^1/2^. This model assumes that hydrodynamic interactions are screened by the segments of overlapping polymeric chains. This mechanism means that the internal segments are shielded, and do not contribute to the viscous drag. On the contrary, the Zimm scaling, G* ~ ω^2/3^, accounts for the hydrodynamic interactions, due to the cooperative motions of internal polymer segments within the system. Then, by analogy with the Zimm model, the results shown in Figure 6 suggest the presence of unshielded internal polymer segments that contribute to viscous drag, with respect to the observed in the pure Rouse model. However, it is problematic to assign these hydrodynamic interactions as in the original Zimm model. It is more likely that the behaviour observed will emerge from a specific distribution of the cPGMA segments at small length scales. A distinct spacing of inter and intramolecular bonds created during the double polymerization process gives rise to a modulation of the interaction of the internal segments with the surrounding nanoassemblies [18]. Then, in addition to the differences observed in their size, a variable density of crosslinking in the cPGMA samples may be anticipated from these results. It should also be noted that the Zimm-like regime extends over decades within the frequency range. This behaviour is a typical response already reported in 3D macromolecules as highly branched dendrimers [13,14,15,16], bottlebrushes [17,18,19,20], highly functionalized stars [21,22,23,24].

It is quite interesting the fact that the terminal zone, especially in the cPGMA1 sample, takes place at very low frequency which indicates a characteristic long relaxation time. This behaviour cannot be explained in the framework of classical polymer dynamics, and it can only be ascribed to a structural relaxation due to the soft colloidal nature of the cPGMA samples [24,25,48]. In this case the relaxation proceeds via a very slow centre-of-mass motions due to the caging of the neighbours. At these low frequencies, the samples are able to relax and the slopes of G′ and G″ reach values close to 2 and 1, respectively.

**Figure 7:**
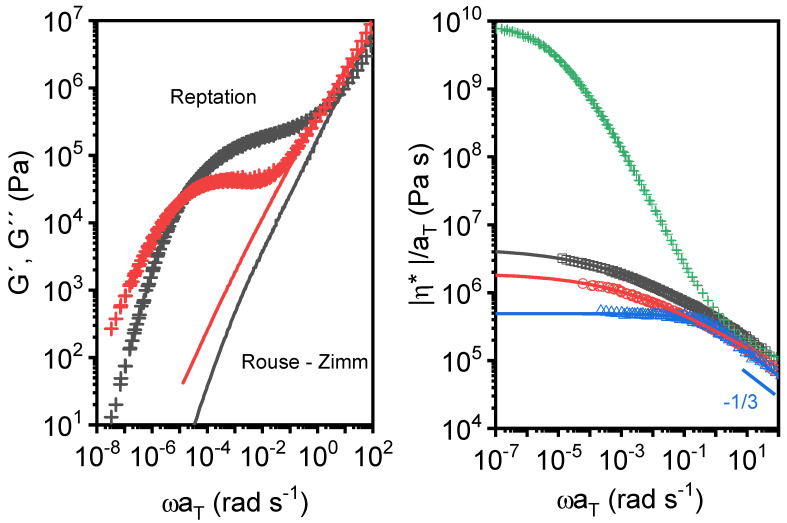
(**A**) Storage and loss moduli master curves of cPGMA3 (lines) and linear PS sample at *T_R_* = *T_g_* + 18 K (391 K). Both samples have the same hydrodynamic size. (**B**) Complex viscosity obtained for the samples under study (☐, black) cPGMA1, (Ο, red) cPGMA2 and (△, blue) cPGMA3 and the (+, green) linear PS sample at *T_R_* = *T_g_* + 18 K (303 K).

The specific construction of the macromolecular assemblies promotes the total absence of entanglements. This phenomenon can be clearly observed in Figure 7A. A direct comparison between the cPGMA1 sample and a polystyrene (PS) sample with identical hydrodynamic radius, *r_h_*, is presented. The temperature has been selected at the same distance as the respective glass transition temperature values for each of the polymers. The relaxation at high frequencies proceeds in a similar way in the samples. However, at a given frequency the PS sample displays a noticeable entanglement plateau, which is a characteristic of a well-entangled polymer melts. This plateau persists on decreasing frequency for 3–4 decades before ultimately incoming the terminal flow at very low frequencies. The cPGMA1 sample shows a power law behaviour that persists over the whole frequency range up to the terminal relaxation. It is important to note that the PS sample has been selected in such a way that the hydrodynamic size is identical to that measured for cPGMA1 sample.

The effect of the absence of entanglements in the samples is also noticeable in Figure 7B, in which the angular frequency dependence of the modulus of the complex viscosity, |η*|, is plotted for the samples in comparison to that obtained for the linear entangled PS reference. The effect of entanglement in PS is clear, and the viscosity at low frequencies reaches a value 3 orders of magnitude higher than those measured in the samples under study at the same distance from *T_g_*. It should be noted that samples exhibit a characteristic regime with slope −1/3 at high frequencies, which matches with the typical scaling predicted by the Zimm model in the complex viscosity curve. We have examined the dependence of the complex viscosity from the angular frequency by fitting the experimental curves to the Carreau model, given by the following expression [49]:

(6) $$\left|\eta^{*}\left(\omega \right)\right|=\frac{\eta_{0}}{{\left[1+{\left(\omega \tau_{0}\right)}^{2}\right]}^{\frac{1-n}{2}}}\;$$

From the fit of Equation (6) to the experimental results in Figure 7B the values of the Newtonian viscosity, *η*_0_, and relaxation time, *τ*_0_, have been extracted. Both parameters, listed in Table 2, decrease when moving from cPGMA1 to cPGMA3. A recent study performed by Luo and co-workers has proposed that the relaxation time (or viscosity) in this type of 3D macromolecular systems depends on both the nanoparticle size and the cross-linking density [50]. The relaxation time increases due to both features at the 30th and 10th power, respectively. Moreover, these authors propose that the soft nanoparticles larger than a certain critical value are not able to relax within the experimental time window available. Our results suggest that the cPGMA samples are well below the critical value, as they effectively relax. Interestingly, the relaxation time measured decreases as the size increases. Considering the recent picture described by Luo and co-workers, the decrease in *η*_0_ and *τ*_0_ may be explained by assuming also a decreased cross-linking density when moving from cPGMA1 to cPGMA3 samples (please note that the molecular weight values in Table 2 are only apparent).

**Figure 8:**
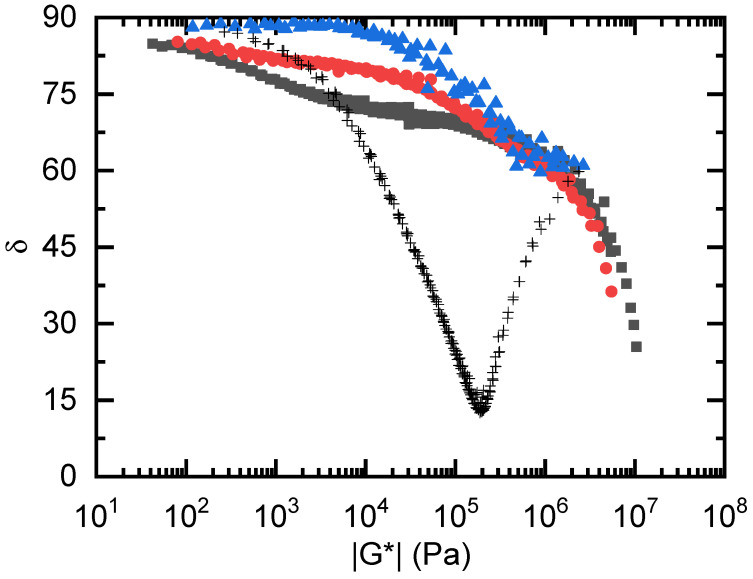
Van Gurp−Palmen plot for (■) cPGMA1, (●) cPGMA2 and (▲) cPGMA3 and (+) linear PS samples.

The van Gurp–Palmen plot is quite useful in rheology, not only for the assessment of the time-temperature superposition, but also to investigate the effect of molecular architecture in polymer dynamics [51]. It has been demonstrated that such a representation is quite sensitive to the molecular parameters (molecular weight distribution) and the topology (branching content and molecular shape) of the polymeric chains [52,53]. In fact, a polymer melt with many distinct relaxation modes will produce a multimodal van Gurp–Palmen plot with different separated local minima due to time/size sequential relaxation mechanisms. Figure 8 presents the linear viscoelastic fingerprint for the samples studied here in comparison to that of the PS reference plotted as the van Gurp–Palmen plot of the phase angle, δ, versus the complex modulus, |G*|. The curves obtained at the different temperatures between 20 °C and 90 °C collapse, for each material, in a single one. This indicates that the cPGMA samples are thermorheologically simple, at least within the temperature range explored. Sample cPGMA3 shows the typical behaviour of an unentangled system, as a unique transition from the glassy dynamics at high values of |G*| to the viscous flow (δ = 90 °) at low values of |G*| is clearly identified. Samples cPGMA2 and cPGMA1 show a characteristic bimodal response, with an inflexion in the intermediate regime just within the zone in which the Zimm-like behaviour is observed for G′ and G″. This specific shape of the van Gurp–Palmen plot has already been observed in systems such as bottlebrushes, wedged and dendronized polymers [18,20,26,27]. The inflexion point marks the onset of the terminal relaxation of the whole system, and it may be attributed to internal relaxation (sliding) of the macromolecular nanoassembly, resulting from the cooperative motions. The van Gurp–Palmen fingerprint of the cPGMA samples is clearly different from the observed in an entangled polymer, for which a sharp minimum in δ function is observed at a characteristic value of |G*| due to the existence of entanglements.

## 4. Conclusions

The rheological properties soft cPGMA nanoassemblies have been determined in a broad range of temperatures, and the time—temperature superposition has been applied to obtain master curves of the whole viscoelastic response from the glassy to the terminal regions. The temperature dependence of the rheological properties follows the free volume WLF theory, but the fragility values obtained are quite lower than the observed in typical amorphous fragile materials. The value obtained is typical of van de Waal fluids, suggesting an effective packing of the macromolecular assemblies. The analysis of the linear viscoelastic fingerprint reveals specific Zimm-like dynamics at intermediate frequencies, as it has been observed in unentangled polymers, special topologies (stars, dendrimers, and bottlebrushes), soft colloids and microgels. All samples show terminal relaxation at low frequencies, suggesting a modulation between the nanoparticle size and the cross-linking density. This is an interesting result as it points towards the possibility of tuning the flow dynamics of the nanoassemblies by means of the polymerization process controlling the size and internal segment density of the nanoparticles.

## Figures and Tables

**Table 1:** Catalysts used for the synthesis of the studied cPGMA

	[AlMeXOAr]	[MAlMeOAr]
cPGMA1	X = Me	M = Li
cPGMA2	X = Cl	M = Li
cPGMA3	X = Me	M = K

**Table 2:** Physical properties of the samples under study: translational diffusion coefficient, D_t_, z-average hydrodynamic size, r_z_, polydispersity, D, number average hydrodynamic size, r_n_, apparent molecular weight, M_w,app_, Newtonian viscosity, η_0_, and relaxation time, τ_0_, and glass transition temperature, T_g_.

Sample	D_t_ (μm^2^/s)	r_z_ (nm) ^a^	Q	r_n_ (nm)	M_w,app_ (kg/mol) ^b^	η_0_ (kPa·s) ^c^	τ_0_ (s) ^c^	T_g_ (K) ^d^
cPGMA1	17.1	23.1	0.40	11.2	800	4488.4	2335	285.5
cPGMA2	8.80	41.1	0.50	20.2	3000	1010.4	23.0	n.d.
cPGMA3	3.60	77.1	0.39	41.4	8000	489.9	0.61	285.0

^a^ Hydrodynamic radius obtained at infinite dilution (c = 0); ^b^ Molecular weight referred to monodisperse PS standards of the same intensity averaged hydrodynamic size in CHCl_3_ at T = 293 K; ^c^ Rheological results obtained at T_r_ = 303 K; ^d^ From references [4,5]; n.d. means not determined.

**Table 3:** Comparison of the catalysts used and the molecular weight of the cPGMA obtained.

	cPGMA1	cPGMA2	cPGMA3
[AlMeXOAr]	X = Me	X = Cl	X = Me
M_w_ (KDa) [3]	33.8	20.0	33.8
[MAlMe_3_OAr]	M = Li	M = Li	M = K
M_w,app_ cPGMA (KDa)	800	3000	8000

## Data Availability

The data presented in this study are available on request from the corresponding author.
